# Psychological Distress in Childbearing Persons During the COVID-19 Pandemic: A Multi-Trajectory Study of Anger, Anxiety, and Depression

**DOI:** 10.1155/da/6663877

**Published:** 2025-03-25

**Authors:** Christine Ou, Guanyu Chen, Gerald F. Giesbrecht, Elizabeth Keys, Catherine Lebel, Lianne Tomfohr-Madsen

**Affiliations:** ^1^Faculty of Human and Social Development, University of Victoria, 3800 Finnerty Road HSD Building, Room A402a, Victoria V8P 5C2, Canada; ^2^Department of Educational and Counselling Psychology and Special Education, University of British Columbia, Neville Scarfe Building, 2125 Main Mall, Vancouver V6T 1Z4, Canada; ^3^Departments of Pediatrics and Community Health Sciences, University of Calgary, 2500 University Drive NW, Calgary T2N 1N4, Canada; ^4^School of Nursing, University of British Columbia Okanagan, ART360 (Arts Building) 1147 Research Road, Kelowna V1V 1V7, Canada

**Keywords:** COVID-19 pandemic, perinatal anger, perinatal anxiety, perinatal depression, trajectories

## Abstract

Psychological distress can manifest as depression, anxiety, and anger in the perinatal period. These conditions are often comorbid yet studied in isolation. A full understanding of perinatal psychopathology requires the spectrum of common psychological distress to be studied concurrently to better understand interconnected symptoms. A transdiagnostic approach provides valuable insights into how symptoms interact and cumulatively affect mental health, which can inform more effective screening and treatment strategies. This, in turn, can improve outcomes for birthing parents experiencing psychological distress. We undertook group-based multi-trajectory modeling (GBMTM) to uncover the patterns of affective disorders (anger, anxiety, and depression) over three-time points (pregnancy, 3-, and 12-months postpartum (mPP)) in a large longitudinal cohort of persons who gave birth during the COVID-19 pandemic (*n* = 2145). We identified five trajectory groups: high-stable (11.3%), postpartum-increase (16.0%), postpartum-decrease (21.5%), low-stable (37.9%), and minimal stable (13.2%) symptoms of anger, anxiety, and depression. Multinomial regression revealed that lower levels of sleep disturbance, less financial hardship, and lower intolerance of uncertainty predicted postpartum decreases in psychological distress compared with the high stable group. Higher levels of sleep disturbance, greater financial hardship, lower level of social support, and greater intolerance of uncertainty predicted postpartum increases in psychological distress compared with the low-stable and minimal-stable groups. Screening for psychological distress symptoms (i.e., anger, anxiety, and depression), paired with access to evidence-based management for those who screen positive, is warranted during the first postpartum year to reduce the harmful effects of unmanaged distress on families.

## 1. Introduction

Persistent psychological distress is a common complication of pregnancy and birth and frequently manifests as symptoms of depression, anxiety, and anger [1–3]. Psychological distress during the perinatal time frame has been linked with adverse birth outcomes such as preterm birth, low-birth weight, increased need for delivery-related interventions [4, 5], and long-term negative outcomes such as impaired child socioemotional development [6, 7]. Unmanaged or undermanaged parental psychological distress can become chronic, leading to lost productivity, intergenerational transmission, and greater needs for services for parents and children, contributing to substantial lifetime economic costs [8–10].

The transdiagnostic theory of mental illness is an integrative framework that identifies core features of mental health disorders and psychological distress [11–13]. Unlike the Diagnostic and Statistical Manual approach of categorizing disorders, a transdiagnostic approach focuses on universal processes such as negative affectivity and emotional dysregulation in mental health disorders [14]. The transdiagnostic framework conceptualizes emotional disorders dimensionally (symptoms on a continuum) rather than categorically (presence or absence) and recognizes that comorbidity of emotional disorders are the norm [13, 14]. Understanding the links between the components of psychological distress can lead to treatment that more fully addresses symptoms to reduce the harmful effects of persistent distress.

Previous studies have primarily focused on anxiety and depression as separate entities [15, 16]. However, recent research suggests that examining these conditions together, including anger, is necessary to provide a more comprehensive understanding of perinatal mental illness [1, 17]. Outside of the perinatal period, evidence indicates that high levels of anger are associated with recurrent, longer-lasting, and more intense depression [18–20] and that individuals with anxiety disorders have greater difficulties with anger [21]. By using a transdiagnostic approach to examining anger, anxiety, and depression in connection with one another, it is possible to capture the complexity of psychological distress and reveal patterns and associations that might be missed when examining each symptom in isolation [14, 22].

The purpose of this study was to examine the trajectories of psychological distress in childbearing individuals who became pregnant during the COVID-19 pandemic using data from the Pregnancy during the Pandemic (PdP) cohort. A pandemic cohort presents an ideal opportunity to investigate psychological distress given the increased prevalence of mental distress symptoms related to lock down measures that reduced social support [23]. This study aimed to: (1) characterize the trajectories of psychological distress (i.e., anger, anxiety, and depression) in childbearing persons from pregnancy through the first postpartum year and (2) identify risk factors that forecast the development of psychological distress using the biopsychosocial model as a framework. We included an examination of sleep quality as a biological variable known to modulate mood and mental health [24, 25]. Psychological trait variables of interest include resilience and intolerance of uncertainty as well-documented influencers of psychological distress [26, 27]. Social and interpersonal variables known to influence perinatal mental health include financial hardship [28], social support [29], and relationship satisfaction [30].

## 2. Materials and Methods

The PdP cohort study was designed to investigate the longitudinal associations between stressors in pregnancy and child developmental outcomes during the pandemic; this pan-Canadian study was launched in April 2020 [31]. The PdP cohort is the largest and earliest recruitment of pregnant Canadians during the COVID-19 pandemic [31]. Data collected included measures of maternal exposure to objective stressors, psychological distress, and risk and protective factors with ongoing data collection continuing for 4+ years after birth to assess maternal mental health (depression, anxiety, and anger) and child development [31].

### 2.1. Design

We conducted a group-based multi-trajectory modeling (GBMTM) analysis of a prospective longitudinal cohort study [31]. GBMTM is a person-centred method for identifying groups within a defined population via concurrent consideration of relevant variables to reveal patterns in the data [22]. Using GBMTM analysis can assist with investigating divergent trends within subgroups of participants, which facilitates the prioritization and tailoring of appropriate screening and intervention protocols for different groups [22].

This analysis is based on data collected between April 5, 2020 and Dec 24, 2022, which occurred during pregnancy (<35 weeks gestational age), 3-, and 12-months postpartum (mPP). The original study received ethical approval (REB20-0500) from the University of Calgary Conjoint Health Research Ethics Board March 26, 2020.

### 2.2. Participants

Eligible participants were at least 17 years of age, less than 35 weeks gestational age at time of enrollment, living in Canada, and able to read and write in English. There were no other eligibility criteria. All participants voluntarily agreed to participate and signed an electronic informed consent form before providing data. The present analysis was restricted to participants who completed all anger-, depression-, and anxiety-related assessments at the aforementioned three-time points (*n* = 2135).

### 2.3. Procedures

Participants were recruited via ads on Facebook and Instagram in both French and English. We used Research Electronic Data Capture (REDCap) platform for participant enrollment, consenting, and administration of surveys.

### 2.4. Measures

#### 2.4.1. Outcome Variables

##### 2.4.1.1. Anger

The presence and intensity of anger in the past week was measured using the Patient Reported Outcomes Measurement Information System (PROMIS) Anger five-item Short-Form (PAS) [32]. Raw scores were converted to *t*-scores for analysis [33]; *t*-scores less than 55 indicate minimal (no) anger; *t*-scores 55–59.9 indicate mild anger; *t*-scores 60–69.9 indicate moderate anger, and *t*-scores ≥ 70 indicate severe anger [33]. The PAS has been validated for use in large samples of adults in clinical and nonclinical settings [32, 33]. In our study, Cronbach's *α* coefficients for the PAS were 0.88 at T1, 0.90 at T2, and 0.90 at T3.

##### 2.4.1.2. Anxiety

Symptoms of anxiety in the past week were measured using PROMIS Anxiety Adult seven-item Short-Form. Raw scores were converted to *t*-scores for analysis; [33] *t*-scores follow the same cutoffs for PAS [33]. Cronbach's *α* coefficients for the PAS were 0.93 at T1, 0.93 at T2, and 0.92 at T3.

##### 2.4.1.3. Depression

Symptoms of depression in the past week were evaluated using the Edinburgh Postnatal Depression Scale (EPDS) [34]. The EPDS has been extensively validated against the Structured Clinical Interview and other diagnostic interviews used for birthing people during pregnancy and postpartum [35, 36]. We used a cutoff ≥13 to indicate possible depression [37]. Cronbach's *α* coefficients for the EPDS were 0.87 at T1, 0.88 at T2, and 0.87 at T3.

#### 2.4.2. Predictor Measures

##### 2.4.2.1. Sleep Disturbance

Sleep quality was assessed over the past 7 days, including difficulties with getting to sleep, overall sleep quality, and restorative sleep, measured by self-reported PROMIS short form Sleep Disturbance 4a with four items. Higher scores indicate greater sleep disturbance. The PROMIS Sleep Disturbance scale has been validated for use in community samples with and without self-reported sleep problems [38]. Raw scores were converted to *t*-scores for analysis, with Cronbach's *α* coefficients of 0.83.

##### 2.4.2.2. Social Support

Perceived interpersonal social support during pregnancy was evaluated using the Interpersonal Support Evaluation List (ISEL) to determine appraisal (e.g., advice or problem solving), belongingness (e.g., shared experiences), and tangible support (e.g., help with daily chores) [39]. Each subscale has four items, and overall social support is calculated as the sum of the three subscales, with higher scores indicating more interpersonal support [39]. The ISEL-12 has been validated for use in U.S. adults [39]. The Cronbach's *α* coefficient for the ISEL was 0.85.

##### 2.4.2.3. Coping and Resilience

Participants' perceived capacity to manage stressful situations was evaluated using the two-item Connor-Davidson Resilience Scale (CD-RISC-2). This scale has strong test–retest reliability and convergent and divergent validity [40]. Each item was scored on a five-point Likert scale, ranging from 0 (“Not true at all”) to 4 (“True all the time”). Total scores ranged from 0 to 8, with higher scores reflecting greater coping success. The Cronbach's *α* for the CD-RISC-2 was 0.66.

##### 2.4.2.4. Uncertainty Intolerance

The 12-item version of the Intolerance of Uncertainty Scale (IUS) was used to assess the degree to which participants perceived uncertainty as stressful, negative, unfair, and inhibiting action [41, 42]. Items were rated on a scale from 1 (“Not at all characteristic of me”) to 5 (“Entirely characteristic of me”) with higher scores indicating greater intolerance of uncertainty. Previous research indicates that the IUS has excellent internal consistency (Cronbach *α* = 0.91) and strong test–retest reliability (*r* = 0.74). It is also closely associated with symptoms of generalized anxiety disorder [41]. The Cronbach's *α* for the IUS was 0.89.

##### 2.4.2.5. Relationship Quality

Participants' perceptions of partner relationship quality were measured using the four-item Couple Satisfaction Index (CSI-4) [43]. Total scores range from 0 to 21, with higher scores reflecting greater relationship satisfaction. CSI-4 scores below 13.5 indicate significant relationship dissatisfaction. The Cronbach's *α* for the CSI-4 was 0.90.

##### 2.4.2.6. Covid Financial Hardship

This predictor variable was created by assessing the impacts of the COVID-19 pandemic on participants' financial circumstances by the question: “Have changes in your personal financial situation during the COVID-19 pandemic made it hard for you to pay for the basics like food, housing, medicine, and/or heating?” Responses were recorded on a scale of 0–100, where “0′ represents not hard at all and “100′ represents very hard. The scale was normalized to a continuous range of 0–1, where 0 represented “Not hard at all” and 1 represented “Very hard.”

### 2.5. Statistical Analysis

#### 2.5.1. Descriptive Analysis

Descriptive statistical analyses were conducted using R (version 4.3.0). Demographic variables, that is, income, education level, planned pregnancy status, ethnicity, relationship status, parity, and COVID-19-related financial hardship, by either proportions or means, are summarized in Table 1. Psychological distress (outcome) variables, included anger, anxiety, and depression at each time point, were summarized by means and ranges, along with the percentages of participants exceeding clinical cutoffs, are presented in Table 2. Means and ranges for the biopsychosocial variables are reported in Table 2.

#### 2.5.2. Trajectories of Postpartum Psychological Distress

Analyses were conducted using Mplus version 8.9. We followed the Guidelines for Reporting Latent Trajectory Studies [44] in reporting our findings. Group-based-trajectory modeling (GBMTM) was used to identify latent participant groups and to characterize the subgroups' profiles [22]. GBMTM allows for the concurrent modeling of multiple measures (i.e., anger, anxiety, and depression) to represent the underlying construct of psychological distress using a finite set of different polynomial functions [22]. GBMTM not only provides the average trajectory for each subgroup but also calculates the probability of each participant's membership in these subgroups. Each individual is allocated to the trajectory group where they have the highest likelihood of belonging. Missing values were managed by full information maximum likelihood estimation [22, 45]. To achieve model robustness, we utilized 500 random sets of starting values and performed 10 optimizations in the final stage [45].

To determine the trajectory forms, we initially explored growth mixture modeling (GMM), which allows random growth factors, such as intercepts and slopes, to vary within each group. However, this approach was unsuitable because of the emergence of a nonpositive definite covariance matrix, challenging the assumption of within-group variation. Consequently, we opted for fixed intercepts and slopes. We considered nonlinear relationships by testing quadratic terms, but none of the nonlinear models were statistically significant. Thus, our final analysis incorporated only fixed intercepts and linear slopes with multiple outcomes.

To identify the optimal number of groups, our analysis began with a one-group model for evaluating the model fit for a single trajectory. The results from the one-group model indicated a poor model fit (RMSEA (90%) = 0.196; CFI/TLI = 0.884/0.768; SRMR = 0.106), with significant variances in growth factors, suggesting the presence of multiple groups. Subsequently, models with two to six groups were compared to find the optimal number of groups with the best model fit indices. Additionally, the parsimony of models, the sample size of the smallest group, and the interpretation of each group were also considered.

#### 2.5.3. Investigating Predictors of Trajectory Membership

We employed the three-step approach [46, 47] to investigate the predictors of distress trajectory group membership. This method first identified latent groups of psychological distress without the impact of covariates. Multinomial regression was then performed to predict a nominal dependent outcome with more than two categories (i.e., latent groups of psychological distress). The group with the lowest levels of distress was selected as the reference group. The coefficients for the other latent groups demonstrated how predictors influenced the likelihood of belonging to a particular group in comparison to the reference group. Our analysis explored the impact of sleep disturbance, tolerance of uncertainty, resilience, financial hardship, availability of social support, and couples' relationship satisfaction as predictors of group membership. To facilitate interpretation, we standardized scores of the measures due to differences in their scales. Odds ratios (ORs) and corresponding 95% confidence intervals (CIs) were calculated for the multinomial regression.

## 3. Results

### 3.1. Descriptive Analysis

Participant sociodemographic characteristics can be found in Table 1. The majority were white (88.0%), in a partnered relationship (98.0%), had an undergraduate degree (79.9%), and reported annual household incomes of $70,000 CAD or higher (88.7%). 59.1% of the participants were expecting their first child. Regarding the impact of COVID-19 on their personal financial situation, the average response suggested minor hardship (mean = 0.14, median = 0.07), from 0 (indicating “not hard at all”) to 1 (“very hard”).

Table 2 shows trends in psychological symptoms over time. Anger symptoms initially increase but then decrease, anxiety symptoms consistently decrease, and depressive symptoms notably decrease from pregnancy to 12 mPP. Correlations among variables of interest at baseline and their changes over time are detailed in Tables S1 and S2 in the Supporting Information.

### 3.2. Trajectories of Postpartum

We fitted GBMTM with fixed intercepts and slopes without within-group variance as discussed in the statistical analysis section. To find the optimal number of groups, Wickrama et al. [48] outline three categories of statistical indices to evaluate model fit: (1) information criteria statistics, including Bayesian information criterion (BIC) and the sample-size adjusted BIC (SSABIC); (2) entropy values; and (3) the adjusted Lo–Mendell–Rubin likelihood ratio test (LMR-LRT) accounting for the sample size [49]. Lower BIC and SSABIC values indicate a better model fit [50]. Entropy is a standardized index (ranging from 0 to 1) that measures classification performance, with higher values indicating clearer separation between classes. Solutions with greater class separation are preferred, as they better differentiate individuals. As per Clark and Muthèn [51], entropy values of 0.40, 0.60, and 0.80 reflect low-, medium-, and high-class separation, respectively. The adjusted LMR-LRT compares the *k*−1 group model and the *k*-group model [52], and statistically significant *p*-values indicate that the *k*-group model (i.e., the current model) provides a better fit than the *k*−1 group model (i.e., a model with one fewer classes) [53, 54].

**Figure 1:**
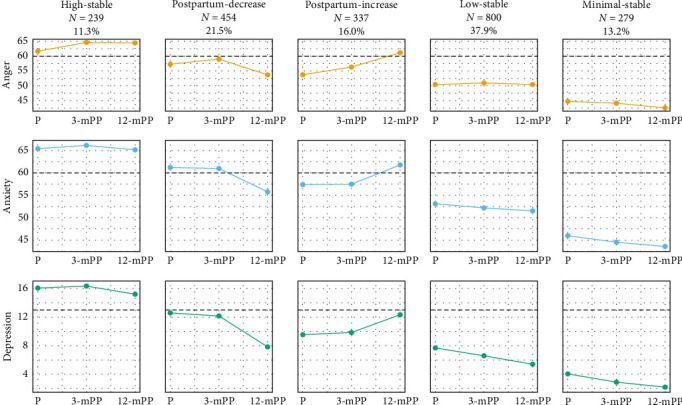
Trajectories of Psychological Distress. EPDS, Edinburgh Postnatal Depression Scale; mPP, months postpartum; P, prenatal. Anger: Patient Reported Outcomes Measurement Information System (PROMIS) Anger Scale; Anxiety: PROMIS Anxiety Scale. Dotted lines represent clinical cutoffs for PROMIS anger or anxiety *t*-scores (≥60), and dashed lines represent clinical cutoffs for EPDS scores (≥13).

Table 3 illustrates that as the number of groups increased, both BIC and SSABIC decreased, but improvement of BIC and SSABIC was accompanied by a decrease in entropy, a nonsignificant adjusted LMR-LRT, and a lower proportion of participants in the smallest group. Specifically, transitioning from the five-group to the six-group model resulted in a nonsignificant Adj. LMR-LRT, with the smallest group's sample size nearing 5%. These results indicated that the six-group model did not provide a statistically significant improvement over the five-group model and compromised interpretability. Therefore, we selected the five-group model of psychological distress with adequate group sizes and entropy value. This five-group model offered trajectories that were clinically relevant and easily interpretable, as shown in Figure 1. There were five distinct groupings: 11.3% of participants exhibited a high-stable pattern of psychological distress, 16.0% were categorized as the postpartum-increasing group, 21.5% showed a postpartum-decreasing trend, the largest cohort of 37.9% maintained a low-stable profile, and 13.2% fell into the minimal-stable category. A detailed description of each group's trends and estimated growth factors can be found in the trajectory group descriptions, Table S3, and Figure S1 in Supporting Information.

### 3.3. Predicting Postpartum

The multinomial regression analysis examined predictors of trajectory group membership, using ORs to compare groups. We examined two aspects of changes in psychological distress: (1) understanding why some individuals experience a decrease in psychological distress from initially high levels (i.e., postpartum-decrease group), compared to others who maintain consistently high levels (i.e., high-stable group) and (2) exploring why some individuals show an increase in psychological distress from initially low levels (i.e., postpartum-increase group) in contrast to those who exhibit minimal changes (i.e., the minimal-stable groups). The complete results of the multinomial regression are available in Table S4 in the supporting Information.

To identify factors that mitigate psychological distress, we compared the high-stable group (as reference group) and postpartum-decrease group in Table 4. We found that primiparous participants had 1.54 times higher odds of being in the postpartum-decrease group as compared to the high-stable group (*p* < 0.05). Participants experiencing severe financial hardship during the COVID-19 pandemic had 0.30 times lower odds of falling into the postpartum-decrease group compared to the high-stable group (*p* < 0.01). Moreover, an increase of one standard deviation in sleep disturbance decreased the odds of being in the postpartum-decrease group by an OR of 0.57 compared to the high-stable group (*p* < 0.001). Higher uncertainty intolerance was associated with 0.71 times lower odds of being in postpartum-decrease group compared to the high-stable group (*p* < 0.01). In summary, primiparous participants, without significant financial hardship, with lower levels of sleep disturbance and lower intolerance of uncertainty, were more inclined to exhibit a decrease in psychological distress, transitioning from a higher to a lower distress level.

Additionally, examining minimal-stable versus the postpartum-increase group identified factors aggravating psychological distress, as illustrated at Table 5. Participants enduring financial hardship during the pandemic had significantly lower odds of being in the minimal-stable group (0.10, *p* < 0.01) compared to postpartum-increase group. An increase of one standard deviation in sleep disturbance resulted in lower odds of 0.59 (*p* < 0.001) for being in the minimal-stable groups compared to postpartum-increase group. Similarly, an increase by one standard deviation in the intolerance of uncertainty contributed to odds of 0.44 (*p* < 0.001) for being in the minimal-stable groups compared to postpartum-increase group. In contrast, an increase in social support was associated with higher odds of 1.62 (*p* < 0.001) for being in the minimal-stable groups compared to postpartum-increase group. Similar patterns were observed when comparing the low-stable group to the postpartum-increase group presented in Table S4 in the supporting information. To recap, financial hardship, increased sleep disturbance, and greater uncertainty intolerance, along with reduced social support, correlated with increasing psychological distress.

## 4. Discussion

In this study of persons who gave birth during the pandemic, we identified five trajectory groups of psychological distress: high-stable (11.3%), postpartum-decrease (21.5%), postpartum-increase (16.0%), low-stable (37.9%), and minimal stable (13.2%) levels of anger, anxiety, and depression. Factors significantly linked with postpartum decreases in psychological distress compared with maintaining high-stable symptoms group included primiparity, low levels of financial hardship and sleep disturbance, and greater tolerance of uncertainty. Factors that were significantly linked with postpartum increases in psychological distress compared with low symptom groups included greater financial hardship, sleep disturbance, and intolerance of uncertainty as well as reduced social support.

Our findings clearly demonstrate the co-occurring nature of the symptoms of psychological distress. This is in line with Cochran et al. [55] findings that irritability, sadness, and worry were transdiagnostic markers of depression across six-time points during pregnancy and the first postpartum year for women with prior psychiatric history. Similarly, Miller and O'Hara [56] found the factor structure of perinatal psychological distress to include symptoms of depression, generalized anxiety, irritability, and panic. Outside of the perinatal time frame, the Netherlands study of depression and anxiety identified anger attacks, depression, and anxiety as comorbid conditions [57].

While depression and anxiety are widely recognized affective disorders, anger is often overlooked as an important aspect of psychological distress. A growing body of research has identified persistent anger as a component of perinatal mental illness for women [1, 2, 58–61] and men [62–64]. Although empirical study has demonstrated that anger is an emotion experienced by men and women equally often, entrenched gender norms affect anger expression and regulation [65]. Anger is stereotyped as a masculine emotion and women's anger expression is often seen as unfeminine and unmaternal because of pervasive ideals about selfless and intensive mothering. Women are more likely to suppress anger [66], and this has been linked with internalizing behaviors such as nonsuicidal self-injury and disordered eating [67]. For some, expressing anger can come with interpersonal costs; persistent anger expressed towards partners can foster conflict and undermine partner support [68, 69], while anger expressed towards children can impair attachment and have negative developmental consequences [70, 71]. For others, the suppression of anger can result in unmet needs and greater depressive symptomology [72]. While depression and anxiety are more routinely screened for, clinicians often do not screen for anger [61, 73]. Parents may not volunteer experiences of anger because of stigma and fears about child apprehension [17], thus, parents with persistent anger may not receive support around managing anger in concert with depression or anxiety.

Multinomial regression revealed that financial hardship was a pregnancy risk factor that predicted increased psychological distress group membership. This is in line with extant findings that income level is a risk factor for perinatal mood disorders. Data from the U.S. 2009–2011 Pregnancy Risk Assessment Monitoring System (PRAMS) of 91,253 women indicated that financial stressors were significantly predictive of postpartum depression and that the odds of experiencing depression symptoms decreased with greater socioeconomic autonomy [74]. In an Australian sample of 1070 postpartum women, participants were 2.45 times more likely to have comorbid anxiety and depression if they were experiencing financial hardship [75]. Meanwhile, income was a significant predictor of postpartum anger in a Canadian sample of 278 women [2]. It is notable that the Canada Emergency Response Benefit was implemented during COVID-19 to mitigate pandemic-related financial hardship [76]. Other analyses of the PdP cohort data indicate that this program did not appear to decrease psychological symptoms [77], indicating that reducing economic adversity remains a policy priority for improving family wellbeing.

Sleep was also a significant predictor of group membership such that conditions of greater sleep disturbance during pregnancy was a risk factor for high or increasing psychological distress, in line with meta-analytic findings about the relationship between antenatal sleep and perinatal depression [78, 79]. In this sample, low baseline social support was associated with increasing distress group membership. Unsurprisingly, pandemic lockdown conditions reduced available social support for many, exacerbating feelings of isolation and amplifying psychological distress [80]. These findings suggest that addressing sleep problems [24, 81], helping expectant parents to build an effective support network of family, friends, and care-providers who can provide formal and informal support [82, 83], identifying parents at risk of persisting psychological distress, and providing accessible mental health services could prevent and reduce psychological distress during pregnancy and after birth [84–86].

### 4.1. Limitations and Future Directions

This study has several limitations to consider. Relying on self-reported survey data may introduce recall bias. Additionally, while the use of shortened forms of some measures helped to reduce participant response burden, it may also compromise the comprehensiveness of the data collected. Moreover, the study design does not allow for causal inferences. Finally, the majority of the participants were partnered, white, and from mid-to-high socioeconomic backgrounds. This limits the generalizability of the findings to parents who are single-parenting, from diverse ethnic backgrounds, or have less income and education. Considering these limitations, the findings of this study should be interpreted with caution. Future research directions include investigating the patterns of psychological distress across different genders and cultures. Ecological momentary assessments of daily behaviors, events, and their impact on perinatal psychological distress could provide insights into the temporal order of variables.

## 5. Conclusion

In this study, we conducted a GBMTM of psychological distress in women who gave birth during the COVID-19 pandemic. The co-occurrence of anger, anxiety, and depressive symptoms highlights the need for a multidimensional approach to understanding emotional disorders during the perinatal period. Additionally, financial hardship, sleep disturbances, and inadequate social support during pregnancy were identified as key risk factors for psychological distress. These findings underscore the critical need for holistic care approaches that address both the psychological and practical needs of expectant and new parents.

## Figures and Tables

**Table 1:** Sociodemographic characteristics of participants.

Variable	Response	*n*	%
Income			
	Less than $20, 000	10	0.5
	$20,000–$39,999	56	2.6
	$40,000–$69,999	175	8.2
	$70,000–$99,999	339	15.9
	$100,000–$124,999	443	20.7
	$125,000–$149,999	323	15.1
	$150,000–$174,999	316	14.8
	$175,000–$199,999	194	9.2
	$200,000+	279	13.1
Education			
	Less than high school diploma	3	0.1
	High school diploma	95	4.4
	College/trade school	334	15.6
	Undergraduate degree	931	43.6
	Master's degree	529	24.8
	Doctorate degree	93	4.4
	Professional (MD, JD, DDS, ETC)	150	7.0
Planned Pregnancy			
	No	251	11.8
	Yes	1884	88.2
Ethnicity			
	Racialized	257	12.0
	White	1878	88.0
Relationship Status			
	Single	43	2.0
	Partnered	2092	98.0
Parity			
	Primiparous	1261	59.1
	Multiparous	872	40.9
	Missing	2	0.1
	—	—	—
	—	*M* (SD)	Range

COVID-19 Financial Hardship	—	0.1(0.2)	0–1

**Table 2:** Descriptive statistics of mental health outcomes and psychosocial variables.

Variable	Time	*M* (SD)	Range	*n*	% above clinical cutoff
PROMIS Anger					
	Prenatal	53.0 (8.1)	32.9–82.9	2135	17.6%
	3-mPP	54.2 (8.8)	32.9–82.9	2135	23.6%
	12-mPP	53.4 (8.8)	32.9–82.9	2135	20.4%
PROMIS Anxiety					
	Prenatal	56.0 (8.0)	36.3–82.7	2135	31.1%
	3-mPP	55.5 (8.3)	36.3–82.7	2135	29.0%
	12-mPP	54.5 (7.9)	36.3–82.7	2135	25.7%
Depression (EPDS)					
	Prenatal	9.5 (5.0)	0.0–26.0	2135	24.8%
	3-mPP	8.9 (5.1)	0.0–28.0	2135	21.3%
	12-mPP	7.7 (4.7)	0.0–26.0	2135	15.1%
PROMIS Sleep Disturbance	—	52.1 (7.5)	32.0–73.3	2135	—
Social Support (ISEL)	—	40.2 (5.7)	13.0–48.0	2135	—
Uncertainty Intolerance (IUS)	—	30.3 (8.4)	12.0–60.0	2135	—
Resilience (CD-RISC-2)	—	5.9 (1.3)	1.0–8.0	2133	—
Relationship Quality (CSI-4)	—	16.9 (3.6)	0.0–21.0	2113	—

*Note*: Clinical cutoffs: PROMIS Anger and Anxiety *t*-scores ≥ 60; EPDS ≥ 13.

Abbreviations: CD-RISC-2, two-item Connor-Davidson Resilience Scale; CSI-4, four-item Couple Satisfaction Index; EPDS, Edinburgh Postnatal Depression Scale; ISEL, Interpersonal Support Evaluation List; IUS, Intolerance of Uncertainty Scale; mPP, months postpartum; PROMIS, Patient Reported Outcomes Measurement Information System.

**Table 3:** GBMTM model estimation (*N* = 2109).

	BIC	SSABIC	Entropy	Adj. LMR-LRT	Smallest group
1 group	129,407.33	129,359.68	—	—	100%
2 group	121,586.99	121,517.09	0.87	6186.48^*∗∗∗*^	48%
3 group	119,744.45	119,652.31	0.85	1861.38^*∗∗∗*^	21%
4 group	118,998.66	118,884.28	0.84	784.72^*∗∗∗*^	13%
**5 group**	**118,569.40**	**118,432.79**	**0.82**	473.99^**∗**^	**11%**
6 group	118,309.11	118,150.25	0.81	308.13	6%

*Note*: For each model, each classes had an intercept and a slope with variances fixed to zero. Models with the optimal number of classes are bolded.

Abbreviations: Adj. LMR-LRT, adjusted Lo–Mendell–Rubin likelihood ratio test; BIC, Bayesian information criteria; GBMTM, group-based multi-trajectory modeling; SSABIC, sample size adjusted BIC.

*⁣* ^*∗*^ *p* < 0.05.

*⁣* ^*∗∗∗*^ *p* < 0.001.

**Table 4:** Postpartum-decrease versus high-stable using multinomial logistic regression.

Variable	Postpartum-decrease vs. high-stable (Ref)
Income	1.025 (0.914, 1.150)
Parity	1.541 (1.038, 2.290)*⁣*^*∗*^
COVID-19 financial hardship	0.296 (0.126, 0.698)*⁣*^*∗∗*^
(PROMIS) Sleep disturbance	0.565 (0.450, 0.709)*⁣*^*∗∗∗*^
Social support (ISEL)	1.188 (0.990, 1.425)
Uncertainty intolerance (IUS)	0.712 (0.573, 0.884)*⁣*^*∗∗*^
Resilience (CD-RISC-2)	0.947 (0.760, 1.179)
Relationship quality (CSI-4)	1.105 (0.928, 1.315)

*Note:* ORs with 95% CIs are reported. High-stable is the reference group.

Abbreviations: CD-RISC-2, two-item Connor-Davidson Resilience Scale; CI, confidence interval; CSI-4, four-item Couple Satisfaction Index; ISEL, Interpersonal Support Evaluation List; IUS, Intolerance of Uncertainty Scale; ORs, odds ratios; PROMIS, Patient Reported Outcomes Measurement Information System.

*⁣* ^*∗*^ *p* < 0.05.

*⁣* ^*∗∗*^ *p* < 0.01.

*⁣* ^*∗∗∗*^ *p* < 0.001.

**Table 5:** Minimal-stable versus postpartum-increase using multinomial logistic regression.

Variable	Minimal-stable vs. postpartum-increase (Ref)
Income	1.036 (0.928, 1.156)
Parity	0.690 (0.451, 1.057)
COVID-19 financial hardship	0.099 (0.025, 0.386)*⁣*^*∗∗*^
(PROMIS) Sleep disturbance	0.588 (0.474, 0.730)*⁣*^*∗∗∗*^
Social support (ISEL)	1.619 (1.248, 2.099)*⁣*^*∗∗∗*^
Uncertainty intolerance (IUS)	0.440 (0.341, 0.568)*⁣*^*∗∗∗*^
Resilience (CD-RISC-2)	1.525 (1.216, 1.913)*⁣*^*∗∗∗*^
Relationship quality (CSI-4)	1.825 (1.316, 2.531)*⁣*^*∗∗∗*^

*Note*: ORs with 95% CIs are reported. Postpartum-increase is the reference group.

Abbreviations: CD-RISC-2, two-item Connor-Davidson Resilience Scale; CI, confidence interval; CSI-4, four-item Couple Satisfaction Index; ISEL, Interpersonal Support Evaluation List; IUS, Intolerance of Uncertainty Scale; ORs, odds ratios; PROMIS, Patient Reported Outcomes Measurement Information System.

*⁣* ^*∗*^ *p* < 0.05.

*⁣* ^*∗∗*^ *p* < 0.01.

*⁣* ^*∗∗∗*^ *p* < 0.001.

## Data Availability

The data that support the findings of this study are available on request from the corresponding author. The data are not publicly available due to privacy or ethical restrictions.

## Supporting Information

Supporting Information Tables S1 and S2 provide correlations amongst predictor and outcome variables and their changes over time. The Trajectory Group Descriptions describes the patterns of change for each trajectory group. Table S3 provides the estimated growth factors for each trajectory group, while Figure S1 visualizes the corresponding estimated trajectories. Last, Table S4 displays the results of the multinomial logistic regression, structured according to different reference groups.
