# Supplementary material for: Psychological Distress in Childbearing Persons During the COVID-19 Pandemic: A Multi-Trajectory Study of Anger, Anxiety, and Depression
Source: Depress Anxiety. 2025 Mar 25;2025:6663877. doi: 10.1155/da/6663877 (PMC11961284; doi:10.1155/da/6663877)
Supplement: Supporting Information — Tables S1 and S2 provide correlations amongst predictor and outcome variables and their changes over time. The Trajectory Group Descriptions describes the patterns of change for each trajectory group. Table S3 provides the estimated growth factors for each trajectory group, while Figure S1 visualizes the corresponding estimated trajectories. Last, Table S4 displays the results of the multinomial logistic regression, structured according to different reference groups. [file 6663877.f1.docx]

**Supplementary Material**

**Table S1**

*Spearman's Correlation Matrix of Key Variables at Baseline (N = 2111)*

|  | PROMIS Anger | PROMIS Anxiety | Depression (EPDS) | PROMIS Sleep Disturbance | Social Support (ISEL) | Uncertainty Intolerance (IUS) | Resilience (CD-RISC-2) | Relationship Quality (CSI-4) |
| --- | --- | --- | --- | --- | --- | --- | --- | --- |
| PROMIS Anger | - |  |  |  |  |  |  |  |
| PROMIS Anxiety | 0.457 | - |  |  |  |  |  |  |
| Depression (EPDS) | 0.594 | 0.581 | - |  |  |  |  |  |
| PROMIS Sleep Disturbance | 0.390 | 0.328 | 0.402 | - |  |  |  |  |
| Social Support (ISEL) | -0.279 | -0.245 | -0.274 | -0.195 | - |  |  |  |
| Uncertainty Intolerance (IUS) | 0.323 | 0.401 | 0.410 | 0.246 | -0.236 | - |  |  |
| Resilience (CD-RISC-2) | -0.275 | -0.287 | -0.380 | -0.235 | 0.253 | -0.419 | - |  |
| Relationship Quality (CSI-4) | -0.367 | -0.155 | -0.230 | -0.174 | 0.331 | -0.103 | 0.141 | - |

*Note.* Twenty-four participants with missing values were excluded. All correlations are significant at *p* < .001

**Table S2**

*Spearman's Correlation Matrix of Outcome Variables Across Timepoints (N = 2135)*

|  |  | Prenatal | | | 3mPP | | | 12mPP | | |
| --- | --- | --- | --- | --- | --- | --- | --- | --- | --- | --- |
|  |  | PROMIS Anger | PROMIS Anxiety | Depression (EPDS) | PROMIS Anger | PROMIS Anxiety | Depression (EPDS) | PROMIS Anger | PROMIS Anxiety | Depression (EPDS) |
| Prenatal | PROMIS Anger | - |  |  |  |  |  |  |  |  |
|  | PROMIS Anxiety | 0.456 | - |  |  |  |  |  |  |  |
|  | Depression (EPDS) | 0.593 | 0.580 | - |  |  |  |  |  |  |
| 3mPP | PROMIS Anger | 0.489 | 0.460 | 0.405 | - |  |  |  |  |  |
|  | PROMIS Anxiety | 0.392 | 0.609 | 0.497 | 0.630 | - |  |  |  |  |
|  | Depression (EPDS) | 0.396 | 0.558 | 0.519 | 0.680 | 0.800 | - |  |  |  |
| 12mPP | PROMIS Anger | 0.471 | 0.411 | 0.382 | 0.541 | 0.432 | 0.440 | - |  |  |
|  | PROMIS Anxiety | 0.372 | 0.543 | 0.450 | 0.436 | 0.589 | 0.523 | 0.668 | - |  |
|  | Depression (EPDS) | 0.394 | 0.521 | 0.498 | 0.467 | 0.534 | 0.595 | 0.693 | 0.793 | - |

*Note.* mPP: Months postpartum. All correlations are significant at *p* < .001

**Trajectory Groups Description**

The first group (n = 11.3%) was labeled as the ‘high-stable symptoms trajectory.’ This group reported consistently high levels of anger (PROMIS Anger t-score range 61.64–64.62), anxiety (PROMIS Anxiety t-score range 65.14–66.13), and depression (EPDS score range 15.22–16.36) from pregnancy to the postpartum period. The second group (n = 16.2%) was described as the ‘postpartum-increase symptoms trajectory.’ This group reported increasing levels of anger (PROMIS Anger t-score range: 53.72–61.15), anxiety (PROMIS Anxiety t-score range: 57.38–61.78), and depression (EPDS score range: 9.55–12.35). The third group (n = 21.8%) was identified as the ‘postpartum-decrease symptoms trajectory.’ Participants in this group experienced decreasing levels of anger (with an increase only at 3 months postpartum; PROMIS Anger t-score range: 53.69–58.99), anxiety (PROMIS Anxiety t-score range: 57.38–61.20), and depression (EPDS score range: 7.84–12.60). The fourth group (n = 37.0%), ‘low-stable symptoms trajectory,’ comprised of the participants, characterized by low levels of anger (PROMIS Anger t-score range: 50.41–50.95), anxiety (PROMIS Anxiety t-score range: 51.52–53.10), and depression (EPDS score range: 5.41–7.69). Lastly, the fifth group (n = 13.6%) was labeled as the ‘minimal-stable symptoms trajectory.’ Participants in this group exhibited minimal symptoms of anger (PROMIS Anger t-score range: 42.56–44.75), anxiety (PROMIS Anxiety t-score range: 43.58–45.98), and depression (EPDS score range: 2.19–4.05), and this group was regarded as the reference group for the multinomial regression analysis. The estimated growth factors are presented in Table S3 and Figure S1.

**Table S3**

*Estimated Growth Factors for Each Group*

|  | High-Stable  (Group 1) | Postpartum-Decrease  (Group 2) | Postpartum-Increase  (Group 3) | Low-Stable  (Group 4) | Minimal-Stable  (Group 5) |
| --- | --- | --- | --- | --- | --- |
| Anger |  |  |  |  |  |
| Intercept | 62.62*** | 58.13*** | 54.20*** | 50.77*** | 44.84*** |
| Slope | 0.17 | -0.32** | 0.55*** | -0.02 | -0.17*** |
| Anxiety |  |  |  |  |  |
| Intercept | 65.73*** | 61.50*** | 57.05*** | 52.84*** | 45.82*** |
| Slope | -0.04 | -0.46*** | 0.36*** | -0.11*** | -0.17*** |
| Depression |  |  |  |  |  |
| Intercept | 16.25*** | 12.82*** | 9.49*** | 7.41*** | 3.82*** |
| Slope | -0.09 | -0.40*** | 0.22* | -0.17*** | -0.13*** |

*Note*. **p*<.05, ***p*<.01, ****p*<.001.

**Figure S1**

*Estimated Growth Lines for Each Group*


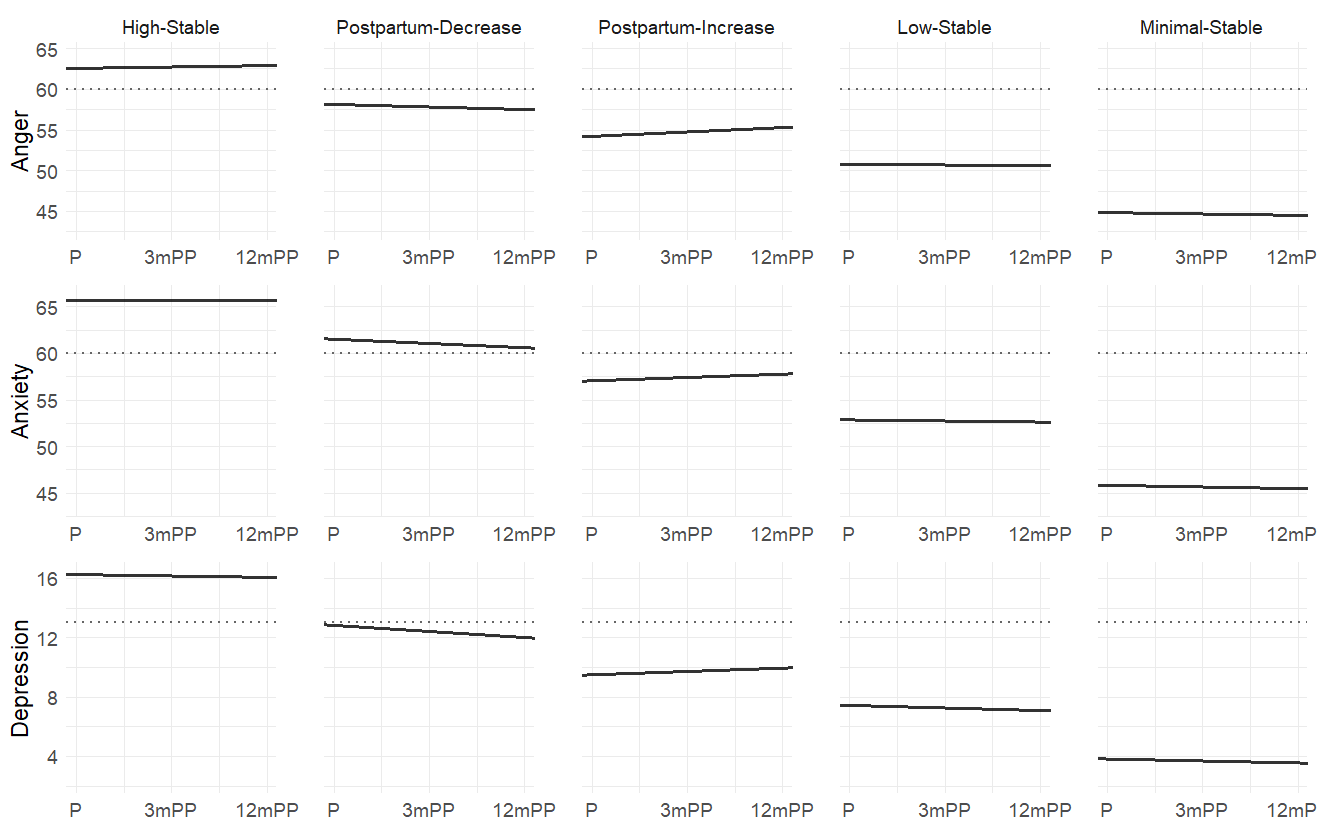


*Note.* P = Prenatal; mPP = Months Postpartum. Anger: PROMIS Anger Scale; Anxiety: PROMIS Anxiety Scale; EPDS: Edinburgh Postnatal Depression Scale. Dotted lines represent clinical cut-offs for PROMIS Anger or Anxiety t-scores (≥ 60), and dashed lines represent clinical cut-offs for EPDS scores (≥13).

**Table S4**

*Test of Predictors for Trajectory Groups using Multinomial Logistic Regression*

|  |  | High-Stable  (Group 1) |  | Postpartum-Decrease (Group 2) |  | Postpartum-Increase  (Group 3) |  | Low-Stable  (Group 4) |  | Minimal-Stable  (Group 5) |
| --- | --- | --- | --- | --- | --- | --- | --- | --- | --- | --- |
| Income | Group 2 | 1.025 (0.914, 1.150) | Group 1 | 0.976 (0.870, 1.094) | Group 1 | 0.953 (0.837, 1.085) | Group 1 | 0.909 (0.813, 1.017) | Group 1 | 0.920 (0.807, 1.050) |
|  | Group 3 | 1.049 (0.922, 1.195) | Group 3 | 1.024 (0.913, 1.149) | Group 2 | 0.977 (0.870, 1.096) | Group 2 | 0.932 (0.853, 1.018) | Group 2 | 0.943 (0.846, 1.052) |
|  | Group 4 | 1.100 (0.983, 1.230) | Group 4 | 1.073 (0.982, 1.172) | Group 4 | 1.048 (0.957, 1.147) | Group 3 | 0.954 (0.872, 1.045) | Group 3 | 0.966 (0.865, 1.078) |
|  | Group 5 | 1.087 (0.953, 1.240) | Group 5 | 1.060 (0.951, 1.183) | Group 5 | 1.036 (0.928, 1.156) | Group 5 | 0.988 (0.903, 1.082) | Group 4 | 1.012 (0.925, 1.107) |
| Parity | Group 2 | 1.541 (1.038, 2.290)* | Group 1 | 0.649 (0.437, 0.964)* | Group 1 | 0.516 (0.327, 0.816)** | Group 1 | 0.516 (0.347, 0.768)** | Group 1 | 0.748 (0.459, 1.218) |
|  | Group 3 | 1.936 (1.225, 3.061)** | Group 3 | 1.256 (0.824, 1.915) | Group 2 | 0.796 (0.522, 1.214) | Group 2 | 0.796 (0.569, 1.112) | Group 2 | 1.153 (0.752, 1.767) |
|  | Group 4 | 1.937 (1.302, 2.881)** | Group 4 | 1.257 (0.899, 1.756) | Group 4 | 1.000 (0.714, 1.401) | Group 3 | 1.000 (0.714, 1.400) | Group 3 | 1.448 (0.946, 2.217) |
|  | Group 5 | 1.337 (0.821, 2.177) | Group 5 | 0.867 (0.566, 1.329) | Group 5 | 0.690 (0.451, 1.057) | Group 5 | 0.690 (0.480, 0.992)* | Group 4 | 1.449 (1.008, 2.083)* |
| COVID Financial Hardship | Group 2 | 0.296 (0.126, 0.698)** | Group 1 | 3.373 (1.433, 7.939)** | Group 1 | 4.281 (1.472, 12.449)** | Group 1 | 11.360 (4.2, 30.723)*** | Group 1 | 43.265 (10.062, 186.040)*** |
|  | Group 3 | 0.234 (0.080, 0.679)** | Group 3 | 0.788 (0.282, 2.203) | Group 2 | 1.269 (0.454, 3.548) | Group 2 | 3.368 (1.338, 8.475)* | Group 2 | 12.827 (3.234, 50.870)*** |
|  | Group 4 | 0.088 (0.033, 0.238)*** | Group 4 | 0.297 (0.118, 0.747)* | Group 4 | 0.377 (0.151, 0.939)* | Group 3 | 2.653 (1.065, 6.611)* | Group 3 | 10.106 (2.592, 39.407)** |
|  | Group 5 | 0.023 (0.005, 0.099)*** | Group 5 | 0.078 (0.020, 0.309)*** | Group 5 | 0.099 (0.025, 0.386)** | Group 5 | 0.263 (0.070, 0.988)* | Group 4 | 3.809 (1.012, 14.334)* |
| (PROMIS) Sleep Disturbance | Group 2 | 0.565 (0.450, 0.709)*** | Group 1 | 1.770 (1.410, 2.222)*** | Group 1 | 2.201 (1.705, 2.842)*** | Group 1 | 3.086 (2.458, 3.876)*** | Group 1 | 3.744 (2.877, 4.873)*** |
|  | Group 3 | 0.454 (0.352, 0.587)*** | Group 3 | 0.804 (0.630, 1.026) | Group 2 | 1.244 (0.974, 1.587) | Group 2 | 1.744 (1.430, 2.127)*** | Group 2 | 2.115 (1.674, 2.672)*** |
|  | Group 4 | 0.324 (0.258, 0.407)*** | Group 4 | 0.573 (0.470, 0.700)*** | Group 4 | 0.713 (0.597, 0.852)*** | Group 3 | 1.402 (1.174, 1.676)*** | Group 3 | 1.701 (1.371, 2.111)*** |
|  | Group 5 | 0.267 (0.205, 0.348)*** | Group 5 | 0.473 (0.374, 0.597)*** | Group 5 | 0.588 (0.474, 0.730)*** | Group 5 | 0.824 (0.691, 0.983)* | Group 4 | 1.213 (1.017, 1.447)* |
| Social Support (ISEL) | Group 2 | 1.188 (0.990, 1.425) | Group 1 | 0.842 (0.702, 1.010) | Group 1 | 0.841 (0.675, 1.046) | Group 1 | 0.666 (0.543, 0.817)*** | Group 1 | 0.519 (0.391, 0.690)*** |
|  | Group 3 | 1.190 (0.956, 1.481) | Group 3 | 1.001 (0.825, 1.215) | Group 2 | 0.999 (0.823, 1.212) | Group 2 | 0.791 (0.666, 0.940)** | Group 2 | 0.617 (0.476, 0.799)*** |
|  | Group 4 | 1.501 (1.224, 1.841)*** | Group 4 | 1.264 (1.064, 1.502)** | Group 4 | 1.262 (1.057, 1.507)* | Group 3 | 0.792 (0.664, 0.946)* | Group 3 | 0.618 (0.476, 0.801)*** |
|  | Group 5 | 1.925 (1.448, 2.560)*** | Group 5 | 1.621 (1.252, 2.099)*** | Group 5 | 1.619 (1.248, 2.099)*** | Group 5 | 1.282 (1.007, 1.634)* | Group 4 | 0.780 (0.612, 0.994)* |
| Uncertainty Intolerance (IUS) | Group 2 | 0.712 (0.573, 0.884)** | Group 1 | 1.405 (1.131, 1.745)** | Group 1 | 2.073 (1.598, 2.689)*** | Group 1 | 2.797 (2.184, 3.583)*** | Group 1 | 4.707 (3.458, 6.407)*** |
|  | Group 3 | 0.482 (0.372, 0.626)*** | Group 3 | 0.678 (0.546, 0.841)*** | Group 2 | 1.476 (1.189, 1.832)*** | Group 2 | 1.991 (1.638, 2.419)*** | Group 2 | 3.350 (2.578, 4.354)*** |
|  | Group 4 | 0.358 (0.279, 0.458)*** | Group 4 | 0.502 (0.413, 0.610)*** | Group 4 | 0.741 (0.617, 0.891)** | Group 3 | 1.349 (1.123, 1.621)** | Group 3 | 2.271 (1.760, 2.929)*** |
|  | Group 5 | 0.212 (0.156, 0.289)*** | Group 5 | 0.298 (0.230, 0.388)*** | Group 5 | 0.440 (0.341, 0.568)*** | Group 5 | 0.594 (0.471, 0.750)*** | Group 4 | 1.683 (1.334, 2.123)*** |
| Resilience (CD-RISC-2) | Group 2 | 0.947 (0.760, 1.179) | Group 1 | 1.056 (0.848, 1.316) | Group 1 | 0.845 (0.651, 1.098) | Group 1 | 0.806 (0.641, 1.012) | Group 1 | 0.554 (0.422, 0.728)*** |
|  | Group 3 | 1.183 (0.911, 1.536) | Group 3 | 1.250 (0.996, 1.568) | Group 2 | 0.800 (0.638, 1.004) | Group 2 | 0.763 (0.635, 0.915)** | Group 2 | 0.525 (0.416, 0.662)*** |
|  | Group 4 | 1.241 (0.988, 1.559) | Group 4 | 1.311 (1.092, 1.574)** | Group 4 | 1.049 (0.878, 1.255) | Group 3 | 0.953 (0.797, 1.139) | Group 3 | 0.656 (0.523, 0.822)*** |
|  | Group 5 | 1.804 (1.373, 2.371)*** | Group 5 | 1.906 (1.512, 2.403)*** | Group 5 | 1.525 (1.216, 1.913)*** | Group 5 | 1.454 (1.198, 1.764)*** | Group 4 | 0.688 (0.567, 0.835)*** |
| Relationship Quality (CSI-4) | Group 2 | 1.105 (0.928, 1.315) | Group 1 | 0.905 (0.760, 1.078) | Group 1 | 0.937 (0.761, 1.154) | Group 1 | 0.896 (0.743, 1.081) | Group 1 | 0.514 (0.366, 0.720)*** |
|  | Group 3 | 1.067 (0.866, 1.314) | Group 3 | 0.966 (0.792, 1.178) | Group 2 | 1.035 (0.849, 1.262) | Group 2 | 0.990 (0.838, 1.170) | Group 2 | 0.567 (0.411, 0.783)** |
|  | Group 4 | 1.116 (0.925, 1.346) | Group 4 | 1.010 (0.855, 1.194) | Group 4 | 1.046 (0.883, 1.238) | Group 3 | 0.956 (0.808, 1.132) | Group 3 | 0.548 (0.395, 0.760)*** |
|  | Group 5 | 1.947 (1.389, 2.730)*** | Group 5 | 1.763 (1.276, 2.435)** | Group 5 | 1.825 (1.316, 2.531)*** | Group 5 | 1.745 (1.271, 2.396)** | Group 4 | 0.573 (0.417, 0.787)** |

*Note.* The Odds Ratios (ORs) with the 95% Confidence Intervals were provided. Each OR compares the trajectory group specified in the row to its reference group indicated at the column’s header. For instance, an OR of 1.025 at the intersection of "Group 2" in the "High-Stable" column indicates that being in the postpartum-decrease group is 1.025 times as likely compared to being in the high-stable group **p*<.05, ***p*<.01, ***
